# In Situ Biosynthesis of Reduced Alpha Hematite (α-Fe_2_O_3_) Nanoparticles by *Stevia Rebaudiana* L. Leaf Extract: Insights into Antioxidant, Antimicrobial, and Anticancer Properties

**DOI:** 10.3390/antibiotics11091252

**Published:** 2022-09-15

**Authors:** Samar Zuhair Alshawwa, Eman J. Mohammed, Nada Hashim, Mohamed Sharaf, Samy Selim, Hayaa M. Alhuthali, Hind A. Alzahrani, Alsayed E. Mekky, Mohamed G. Elharrif

**Affiliations:** 1 Department of Pharmaceutical Sciences, College of Pharmacy, Princess Nourah bint Abdulrahman University, Riyadh 11671, Saudi Arabia; szalshawwa@pnu.edu.sa; 2 Department of Biology, College of Science, Mustansiriyah University, Baghdad 14022, Iraq; emanjassim@uomustansiriyah.edu.iq; 3 General Practitioner, Faculty of Medicine, University of Gezira, Wad Medani 318, Sudan; hashimnada345@gmail.com; 4 Department of Biochemistry, Faculty of Agriculture, AL-Azhar University, Cairo 11751, Egypt; 5 Department of Clinical Laboratory Sciences, College of Applied Medical Sciences, Jouf University, Sakaka 72341, Saudi Arabia; 6 Department of Clinical laboratory sciences, College of Applied Medical Sciences, Taif University, Taif 21944, Saudi Arabia; hmhuthali@tu.edu.sa; 7 Basic Sciences, Applied Medical Sciences, Albaha University, Albaha 4781, Saudi Arabia; hindalzahrani@bu.edu.sa; 8 Department of Botany and Microbiology, Faculty of Science, Al-Azhar University, Cairo 11884, Egypt; Alsayedessam@Azhar.edu.eg; 9 Department of Basic Medical Sciences, College of Medicine, Shaqra University, Shaqra 11961, Saudi Arabia; al_harrif@yahoo.com

**Keywords:** *Stevia rebaudiana* L., IONP green synthesis, antioxidant, cytotoxic, antitumor, extraction yield

## Abstract

In the present study, we utilized *Stevia rebaudiana* L. (SRLe) extract to in situ biosynthesize nanoscale alpha hematite (α-Fe_2_O_3_) nanoparticles (NPs) with potent antioxidant, antimicrobial, and anticancer properties. SRLe-α-Fe_2_O_3_ was characterized using physiochemical analyses, including UV/Vis, FTIR, XRD, DLS, EDX, SEM, and TEM studies. Among tested solvents, CHCl_3_/MeOH (2:1 *v*/*v*) SRL extract (least polar solvent) contained the highest EY, TPC, and antioxidant capacity of ~3.5%, ~75 mg GAE/g extract, and IC_50_ = 9.87 ± 0.7 mg/mL, respectively. FTIR confirmed the engagement of coating operation to the colloidal α-Fe_2_O_3_ NPs. TEM, SEM, and DLS revealed that SRLe-α-Fe_2_O_3_ has a spherical shape, uniform size distribution with aggregation for an average size of ~18.34 nm, and ζ = −19.4 mV, forming a repulsive barrier that helped to improve stability. The synthesized nanoparticles displayed considerable antibacterial activity against *E. coli* and *S. aureus* bacterial growth, and exhibited superior activity against the A549 lung cancer cell lines. These findings indicate that the increased availability of bioactive substances with antioxidant properties of SRLe makes it a potentially interesting material for the preparation of biologically active compounds and green synthesis of nanoparticles.

## 1. Introduction

**Figure 1:**
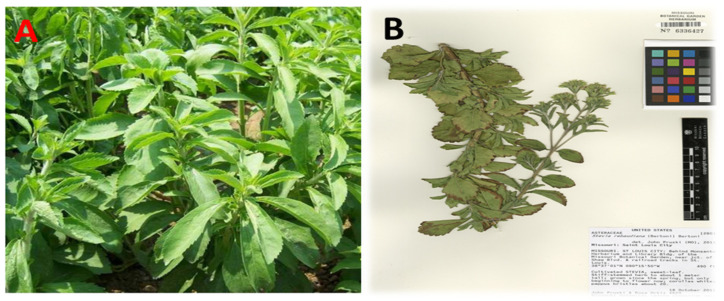
(**A**) *S. rebaudiana (Bertoni) Bertoni Asteraceae* leaves; (**B**) Botanical Voucher Specimen, ↑ MOBOT, Tropicos.org (http://www.tropicos.org/Image/100229514, 1 March 2022).

Plants are a treasure trove of natural bioactive compounds represented in their secondary metabolites and antioxidants [1]. *Stevia rebaudiana* L. (S. *rebaudiana*) is a perennial herb [2]; its leaves have rich contents of phenolic compounds such as phenolic acids, flavonoids, proteins, vitamins, and essential and nonessential fatty acids [3]. Various studies have reported new phenol and polyphenol compounds identified as flavonoids and glycosides in *Stevia* leaves, indicating the importance of the nutritive structure of *S. rebaudiana* (Figure 1) [4]. 

Phenolic compounds are amphipathic molecules, having a peculiar chemical composition with at least one aromatic ring, in addition to one or more attached hydroxyl groups. They have variable molecular weights, with a huge number of subgroups distinguished as flavonoids, chalcones, coumarins, hydroxycinnamic acids, hydroxybenzoic acids, etc. All these families are renowned as having antioxidant and antitumor properties [5,6]. However, hydroxycinnamic acids represent an important group of phenolic compounds derived from cinnamic acid through the phenylpropanoid pathway. Ferulic, caffeic, and *p*-coumaric acids are the most abundant compounds of the biosynthetic pathway in this group. These compounds show broad biological and pharmacological properties, particularly anticarcinogenic, antioxidant, antiviral, and antiallergic activities [7]. 

The availability of bioactive substances with antioxidant/reducing properties makes *S. rebaudiana* a potentially interesting raw material for the preparation of bioactive compounds and green synthesis of nanoparticles [8]. On the other hand, in recent years, magnetite NPs, mainly Fe_3_O_4_, have generated extreme interest in biomedical applications for magnetic separation and resonance imaging, drug delivery, engineering of tissue, tracking of cells, bio-separation, and magnetic hyperthermia [9]. 

Several studies have proven that attached antioxidants on the nanoparticle surface increase antioxidant activity and bioavailability for long periods. Thus, the nanoparticles must have biocompatibility and a high saturation of magnetic and surface interaction [10]. Moreover, Fe_3_O_4_ nanoparticles are comparatively safe, do not accumulate in bio-organs. and are quickly eliminated from the body, as shown by in vivo studies [11]. 

Magnetic IONPs have prominent antioxidant activity against oxidative damage-related diseases [12,13]. However, the antioxidant activity of nanomaterials is strongly influenced by many factors, such as chemical composition, particle size, surface charge, and coating of the surface [14,15]. The surface coating should be nontoxic and biocompatible, enabling delivery of the targeted drug [16,17]. Studies have elucidated that antioxidants attached to the surface of nanomaterials induce antioxidant activity and bioavailability [10,18]. 

**Figure 2:**
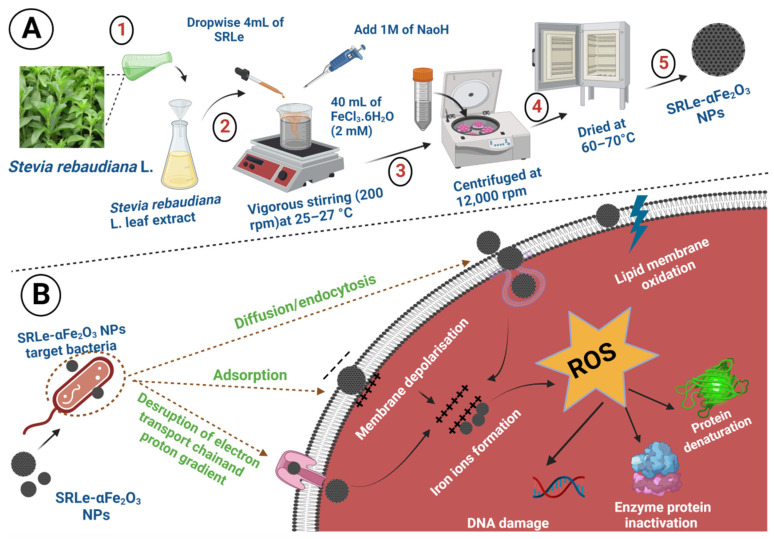
(**A**) Synthesis route of SRLe-α-Fe_2_O_3_ NPs; (**B**) SRLe-α-Fe_2_O_3_ targeting and killing of bacteria via different mechanisms.

Furthermore, the antioxidant activity of either single or bimetallic combination synthesized nanocomposites via chemical or green techniques utilizing various phytochemicals (leaf extracts) was also evaluated [19]. Nanoparticles present many advantages compared to traditional antioxidant delivery methods, which include raising the bioavailability and environmental protection of the bioactive components, targeted delivery of antioxidants, and controlled freeing at the site of action [15]. Engineered nanostructured particles have recently been considered an innovative strategy to provide novel antioxidants with enhanced characteristics. Nanoparticles enhance the natural antioxidant enzyme activity by providing increased target delivery of compounds that show poor permeation across cell membranes and inadequate cell internalization [20]. However, limited studies are available regarding the biological synthesis of nanoscale alpha hematite (α-Fe_2_O_3_) and its application as an antimicrobial and anticancer agent. Hence, this study aims to investigate the size-controlled green synthesis process using SRLe and the surface functionalization synthesis of nanoscale alpha hematite (α-Fe_2_O_3_) NPs via in situ oxidation–precipitation methods, along with the prediction of biological activities, antimicrobial activities, and anticancer. Furthermore, this is the ever first report to elucidate the phytochemicals in *S. rebaudiana* plant extract and to synthesize *S. rebaudiana* leaf extract-mediated α-Fe_2_O_3_ NPs with provden antibacterial and anticancer efficacy. A schematic representation of the present study is clearly depicted in Figure 2.

## 2. Results and Discussion

### 2.1. Effect of Solvent Polarity on Extraction Yield (EY) and TPC of S. rebaudiana L. Extracts (SRLe)

**Figure 3:**
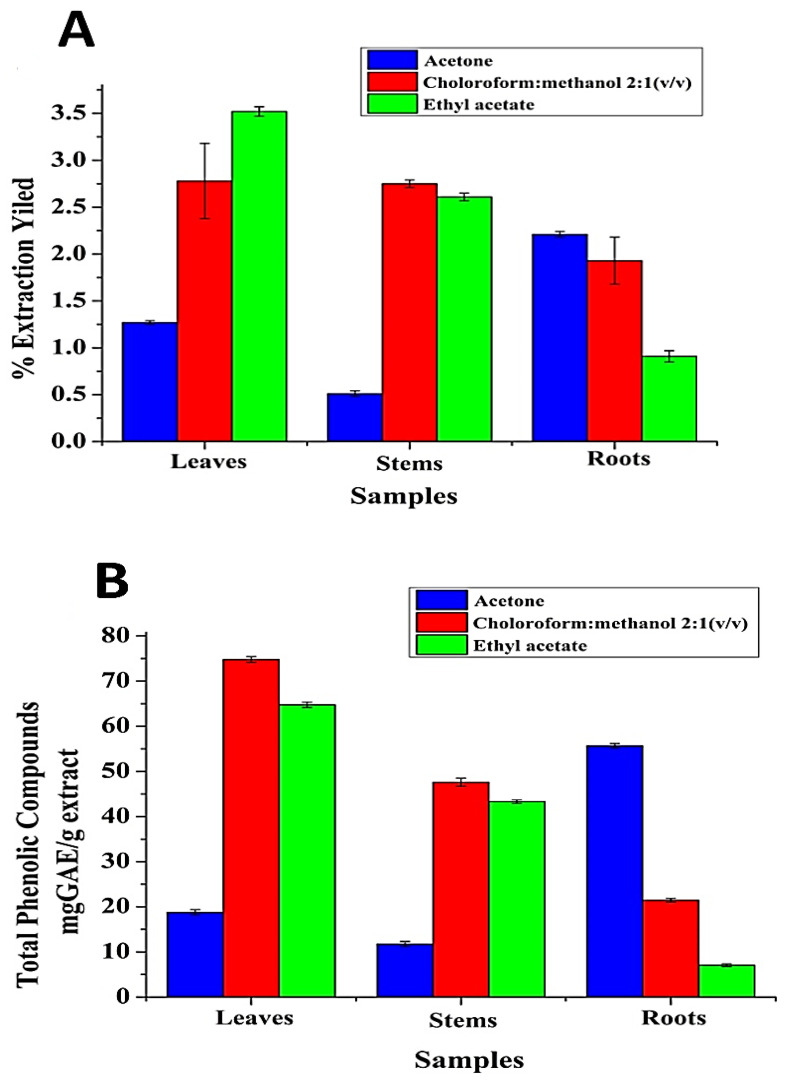

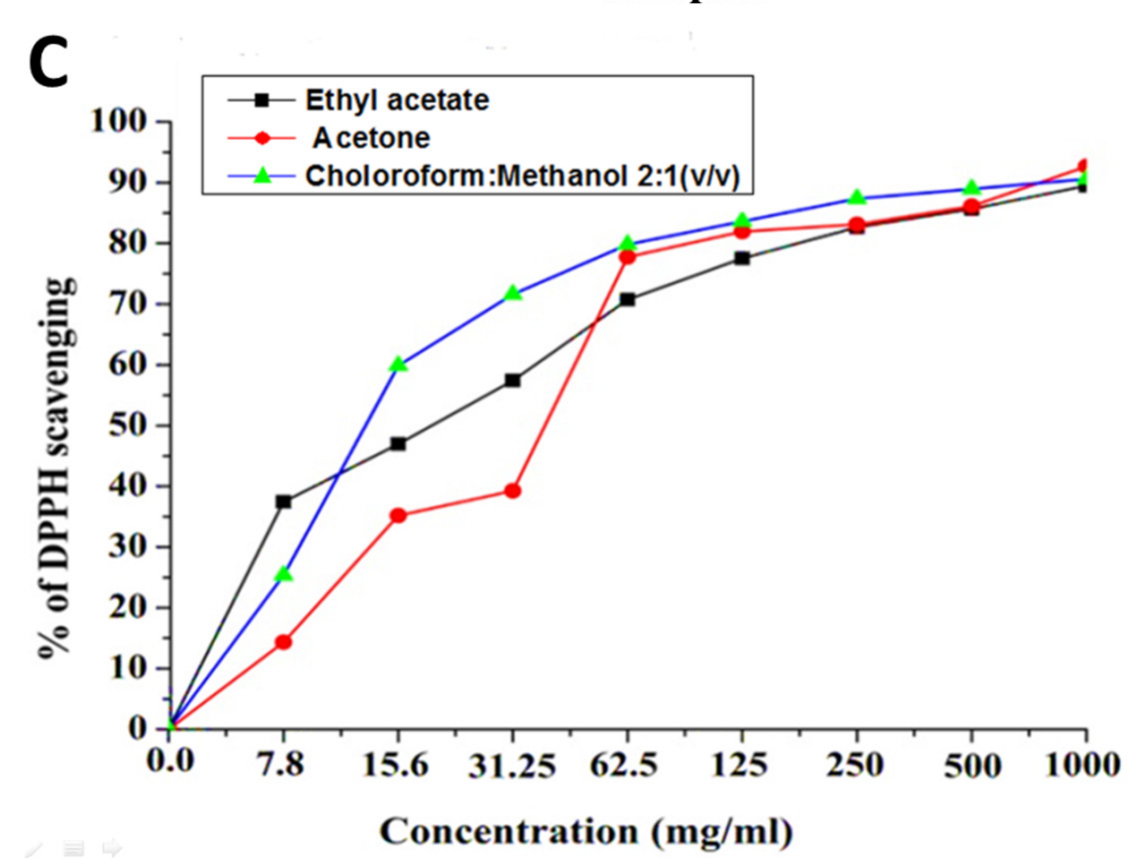
Effect of solvents on the recovery of (**A**) EY and (**B**) TPC of *SRLe*. The values are presented as means ± SD (*n* = 3). (**C**) Scavenging activity on DPPH radicals (%).

It is well known that the EY of bioactive chemical compounds relies on various factors, including the types of solvents with varying polarities, pH, time, and extraction temperature, in addition to the chemical installation of the basic samples. Under the same time and temperature conditions, the solvent and the chemical properties of the sample are the two most important factors [21]. In this study, three solvents were tested to estimate the EY and TPC from various *SRLe* parts (leaves, stems, and roots). The results are shown in Table 1 and Figure 3A, B. Among the tested solvents, CHCl_3_/MeOH (2:1 *v*/*v*) leaf extract (least polar solvent) contained the highest EY and TPC of ~3.5% and ~75 mg GAE/g extract. AcOH root extract (most polar solvent) contained the lowest EY and TPC of ~2.2% and ~55.6 mg GAE/g extract. EtOAc stem extract exhibited an EY and TPC of ~2.7% and ~64 mg GAE/g extract, respectively. These outcomes are consistent with previously reported results by Kim et al. [22]. However, these findings also indicate that the extraction efficiency depends on the polarity of the solvents. The significant modifications in the content of TPC in *Stevia* extract confirmed that the chemical polarity properties of the solvent influenced the qualitative structure and physicochemical activity of the extracts, as confirmed in a previous report by Bęben et al. [23].

### 2.2. Antioxidant Activity

Extraction solvents affect the EY and the TPC, thus significantly affecting the biological activity of the extract [24]. The antioxidant activities of *SRLe* were indexed according to the DPPH radical-scavenging activity (Figure 3C). The inferences showed that all samples possessed varying antioxidant and free-radical-scavenging activities. Among the tested extracts, CHCl_3_/MeOH (2:1 *v*/*v*) leaf extract had significantly higher radical-scavenging activity with an IC_50_ value of 12.87 ± 0.7mg/mL as compared to the EtOAc leaf extract, while AcOH root extract exhibited significantly lower radical-scavenging activity with IC_50_ values of 20.07 ± 1.2 mg/mL and 36.54 ± 1.1 mg/mL, respectively, compared to the control (ascorbic acid, IC_50_ = 11.2 ± 0.6 μg/mL). This behavior is similar to that reported by Criado et al. [25], Furthermore, Ruiz et al. [26] investigated the free-radical-scavenging activity of *S. rebaudiana* extracts at various doses. *S. rebaudiana* extract’s radical-scavenging efficacy improved with increasing dose (25–625 mg/mL). The IC50 value was determined to be 335.94 g/mL. These findings suggest that the CHCl_3_/MeOH (2:1 *v*/*v*) extract of *S. rebaudiana* is a potentially strong antioxidant agent for the improvement of additional drugs. According to Kähkönen et al., *S. rebaudiana* extract may have higher antioxidant activities since it has a larger TPC [27].

### 2.3. Identification of Bioactive Phenolic Compounds of SRLe Using HPLC

**Figure 4:**
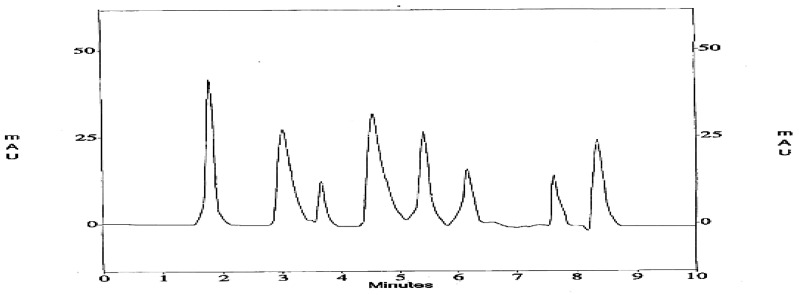
Chromatogram of bioactive phenolic compounds in CHCl_3_/MeOH (2:1 *v*/*v*) SRLe at 245 nm.

HPLC currently represents the most popular and reliable technique for the analysis of phenolic compounds. Various supports and mobile phases are available for the analysis of phenolics including anthocyanin, proanthocyanins, hydrolysable tannins, flavonols, flavan-3-ols, flavanones, flavones, and phenolic acids in different plant extracts and food samples [28]. Under the current conditions, CHCl_3_/MeOH (2:1 *v*/*v*) of *SRLe* had a slightly higher phytochemical content accumulation than stems and roots. The peaks in the HPLC chromatogram of CHCl_3_/MeOH (2:1 *v*/*v*) leaf extract were identified by comparing the retention time and UV spectra of bioactive phenolic compounds in the sample with standards within 10 min. The HPLC results of bioactive phenolic content are listed in Table 2. Eight phenolic compounds were identified in the CHCl_3_/MeOH (2:1 *v*/*v*) extract according to HPLC chromatograms: ferulic acid, syringic acid, protocatechuic, catechin, coumaric acid, caffeic acid, gallic acid, and chlorogenic acid. As indicated in Table 2 and Figure 4, a substantial peaks of gallic acid, syringic acid, and coumaric acid, with concentrations of 13.483 µg/mL, 7.825 µg/mL, and 6.154 µg/mL, respectively, were found. According to Mynit et al. (2020), chlorogenic acids, isochlorogenic acids, and other hydroxycinnamic acids, make up the majority of the potential polyphenols in the leaves of *S. rebaudiana* (Bertoni) [29]. Many bioactive phenolic compounds have also been found in stevia leaves [4,30], presenting potential bioactivities, such as coumaric acid [4], catechin, gallic acid, syringic acid, and caffeic acid [31].

### 2.4. Characterization of SRLe-α-Fe_2_O_3_ Dispersions

#### 2.4.1. Practical Size (PS), PDI, and ζ-Potential

**Figure 5:**
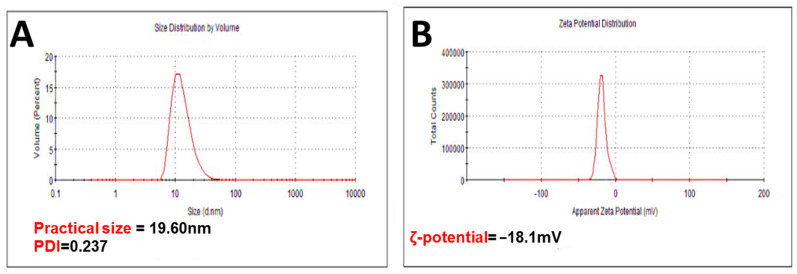
(**A**) Hydrodynamic size and polydispersity index (PDI) of *SRLe*-αFe_2_O_3_ NPs; (**B**) ζ-potential of *SRLe*-αFe_2_O_3_ NPs. Numerical data are reported as the mean ± SD ζ-potential (*n* = 3) and particle size and PDI (*n* = 4).

Dynamic light scattering (DLS) was used to measure the PS, PDI, and ζ-potential of bare SRLe-αFe_2_O_3_. The mean values recorded for all the systems showed a PS distribution in nanometers as shown in Figure 5. The size of *SRLe*-αFe_2_O_3_ was about ~19.60 ± 3.8 nm, while the PDI was 0.237 (Figure 5A). Furthermore, a very narrowly distributed particle possesses PDI values of about 0.01–0.3, which is ideal for stability and uniformity of dispersion [32]. The stability of nanoparticles is generally predicted from their ζ-potential values; here, the ζ-potential value was determined to be −18.1 ± 1.6 mV for *SRLe*-αFe_2_O_3_ (Figure 5B). A ζ-potential value higher than −30 mV is considered to be stable due to electrostatic balance [33]. The negative ζ-potential charge of *SRLe*-αFe_2_O_3_ could be attributed to the ionization of the phenolic hydroxyl groups in the capping moieties at alkaline pH [34], indicating good coating of magnetite iron surface cations through Fe–O linkage [35]. The high negative charge formed a repulsive barrier that helped to avoid the aggregation and improve the colloidal stability of *SRLe*-αFe_2_O_3._

#### 2.4.2. Fourier-Transform Infrared Spectroscopy (FT-IR)

**Figure 6:**
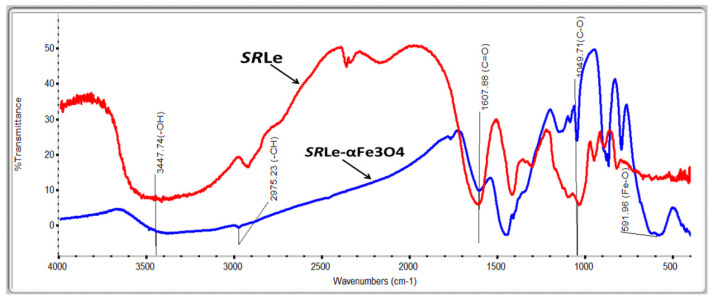
FTIR spectra of *SRL* extract and *SRLe*-αFe_2_O_3_ green synthesis.

The FTIR showed a different vibration according to functional groups with a characteristic absorption in the IR region. FTIR spectroscopy is useful for detecting the characteristic peaks and functional groups of compounds. The FTIR spectrum of the extract is shown in Figure 6. The spectrum showed an –OH band in the frequency range 3200–3600 cm^−1^, C–H stretching in the frequency range 2800–3000 cm^−1^, C=O stretching at the frequency of 1628 cm^−1^, and C–O stretching in the frequency range of 1050–1400 cm^−1^. The presence of the –OH band and C=O stretching suggests that the constituents in the extract had –OH and C=O groups as functional groups. Phenolic and flavonoid compounds are compounds containing hydroxyl and carbonyl groups as functional groups [36]. These annotations affirm the existence of –OH moieties, which are capable of terminating the propagation of chain carrying radicals by acting as an H-atom donor. The C–OH group of phenols was responsible for the peak at 1160 cm^−1^, indicating the presence of polyphenols such as terpenoids and flavonoids, which may also operate as bio-reducing agents. As a result, proteins can serve as both stabilizing and reducing agents. Fe–O nanoparticles were responsible for the peak at 770 cm^−1^. These functional groups were observed on the surface of *SRLe*-αFe_2_O_3_ NPs produced from leaf extracts of stevia plants. These results are in line with previous studies on green nanoparticle synthesis [37].

#### 2.4.3. UV/Visible (UV/Vis) Spectroscopy Analysis

**Figure 7:**
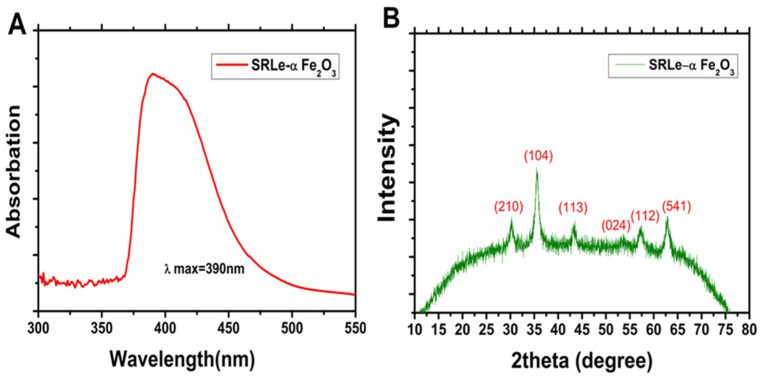
(**A**) UV/visible absorption spectra; (**B**) XRD analysis of *SRLe*-αFe_2_O_3_ NPs.

The coordination complex was validated as the absorption peak was identified at 390 nm using the UV/visible spectral analysis of the synthesized *SRLe*-αFe_2_O_3_ NPs (Figure 7A). Our result coincides with previously published results [38,39]. Additionally, the single and strong peak at 390 nm in the UV/Vis absorption spectrum confirmed that the *SRLe*-αFe_2_O_3_ NPs had a spherical shape, similar to the findings of Kumar et al. (2022) [40].

#### 2.4.4. XRD Analysis

The degree of crystallinity of the synthesized *SRLe*-αFe_2_O_3_ NPs was determined via powder XRD analysis. Figure 7B depicts the powder XRD patterns of *SRLe*-αFe_2_O_3_ NPs with a number of distinctive peaks (2θ) at diffraction angles of 29.42°, 34.04°, 43.95°, 55.12°, 57.46°, and 64.83°, corresponding to indices (210), (104), (113), (024), (112), and (541) on JCPDS card numbers 33-0664 and 341266, matching nicely with the crystal structure of α-Fe_2_O_3_ NPs [39,41].

#### 2.4.5. Surface Properties

**Figure 8:**
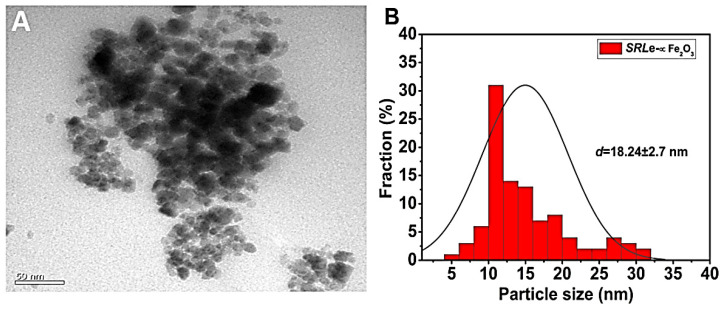
(**A**) Average particle size and size distribution of prepared *SRLe*-αFe_2_O_3_ NPs measured using TEM; (scale bar = 50 nm). (**B**) Size distribution data presented as the mean ± SD (*n* = 3).

**Figure 9:**
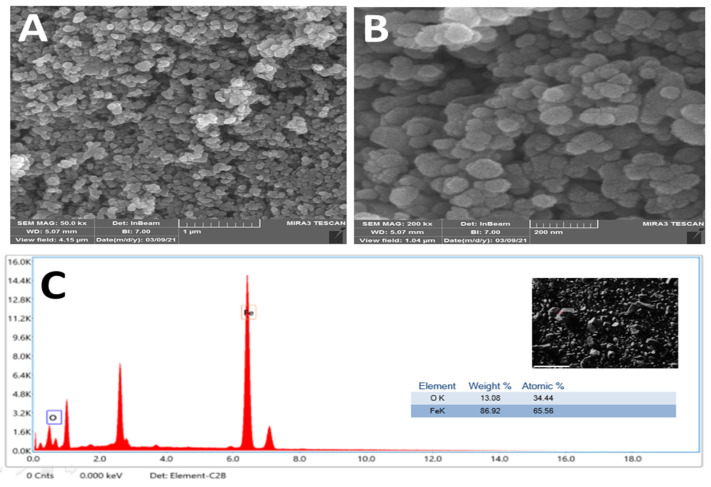
(**A**,**B**) Average particle size of prepared *SRLe*-αFe_2_O_3_ NPs measured using SEM (scale bar = 500 nm in (**A**) and 200 nm in (**B**)). (**C**) EDX profile.

The size distribution and morphology are illustrated in Figure 8 and Figure 9. *SRLe*-αFe_2_O_3_ was analyzed using TEM (Figure 8A,B) and SEM (Figure 9A–C) microscopy images matched to those obtained using DLS. Each TEM and SEM image showed particles with a spherical shape, uniform size distribution, and aggregation. The *SRLe*-αFe_2_O_3_ size distribution for functionalized magnetite NPs was uniform with an average size of ~18 ± 2.7 nm. Similar sizes of green synthesized silver nanoparticles using fresh *Sida cordifolia* extract were reported, with typical size diameters ranging from 15 to 18 nm [42]. Overall, prior results confirm that utilizing aqueous leaf extract of *SRLe* to synthesize alpha hematite nanoparticles is an eco-friendly and high-efficiency approach, as demonstrated in our current investigation.

Using energy0dispersive X-ray spectroscopy (EDX), the elemental mapping of the biogenic *SRLe*-αFe_2_O_3_ was discovered. Oxygen (34.44%) and iron (65.56%) were both detected through EDX analysis, as shown in Figure 9C. Additionally, a strong peak at 6.44 keV was seen, suggesting the presence of iron (Fe), while another peak at 7.02 keV was attributed to the presence of iron (Fe) [43]. According to previous research, several elements discovered through EDX analysis, such as Si, Fe, and Cl, were revealed to behave as capping agents of biogenic αFe_2_O_3_ [44].

### 2.5. Antimicrobial Activity of SRLe-αFe_2_O_3_ NPs According to Agar Well Diffusion Assay

**Figure 10:**
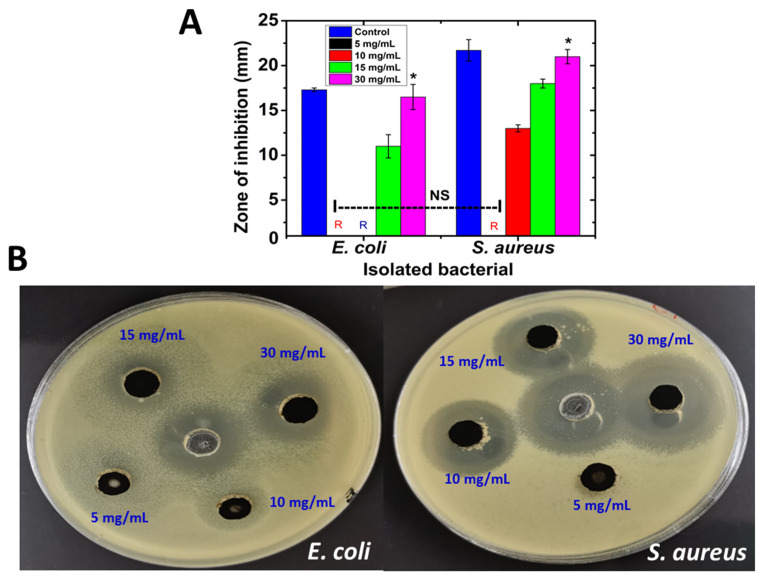
*SRLe*-αFe_2_O_3_NPs inhibit *E. coli* and *S. aureus* bacterial growth. The inhibitory effect of *SRLe*-αFe_2_O_3_NPs compared with erythromycin as a standard antibiotic control was determined in vitro using an agar well diffusion assay. (**A**) Zones of inhibition (mm). Each column shows the mean ± SD of three independent experiments. * Statistically significant difference (*p* < 0.05); NS represents a nonsignificant differences (*p* > 0.05) compared to the control sample. The symbol “R” indicates resistance to the studied bacterial strains. (**B**) Bacterial zones of inhibition (mm) on *E. coli* and *S. aureus* plates with diverse concentrations (5, 10, 15, and 30 mg/mL) of *SRLe*-αFe_2_O_3_NPs.

Green synthesized nanoparticle suspensions of various concentrations were tested for antibacterial activity against *E. coli* and *S. aureus* using the well diffusion method. The ability of the antibacterial agent (NPs) is shown in Figure 10A,B, revealing that the synthesized nanoparticles clearly displayed antibacterial properties. The maximum effect was noted for *S. aureus,* while the minimum effect was noted for *E. coli* in a dose-dependent manner. For *S. aureus*, the zones of inhibition were 13 ± 0.4 mm and 18 ± 0.5 mm for the 10 mg/mL and 15 mg/mL doses, respectively, which almost doubled to an inhibition zone of 21.5 ± 0.8 mm for 30 mg/mL. No inhibition was detected at the 5 mg/mL dose. It has been reported that iron nanoparticles possess antibacterial properties due to their nanoscale size, which enables them to accumulate and deposit on the surface of bacteria during testing [45,46]. Additionally, plant extracts are also likely to possess antibacterial properties due to their high phytochemical content [47]. The activity against both Gram-positive and Gram-negative bacteria of hematite (α-Fe_2_O_3_) NPs can be explained by several possible mechanisms such as stability in an ambient environment, generation of ROS (superoxide radical anions (O^2^−), hydroxyl radicals (OH−), etc.), oxidative stress, and release of ions by nanoparticles reacting with the bacteria’s thiol groups (–SH), which can alter the cellular structure of microorganisms, thus interrupting DNA reproduction and inhibiting enzyme and protein synthesis [48,49]. Furthermore, the NPs exhibited a moderate effect on both *E. coli* strains with inhibition zones of approximately 11 ± 1.3 mm and 16.5 ± 1.4 mm, respectively, at doses of 15 mg/mL and 30 mg/mL. At the same time, no inhibitions were detected at doses of 5 mg/mL and 10 mg/mL. These results were significantly similar to those of Bhuiyan et al. (2020), who reported no antibacterial efficiency for α-Fe_2_O_3_ synthesized using a leaf extract of *Carica papaya* against *E. coli* strains at a concentration of 5 (mg/disc) [50]. A negatively charged bacterial surface might be disrupted and destabilized by the positively charged metal ions released by NPs, resulting in cell death. [51]. As a result, the nanoparticles were shown to have greater effectiveness against Gram-positive bacteria as compared to Gram-negative bacteria, potentially due to Gram-negative bacteria having an extra layer of lipopolysaccharide and peptidoglycan on top of their cell walls, granting them the ability to resist the damage caused by nanoparticles.

### 2.6. Cytotoxicity Study

**Figure 11:**
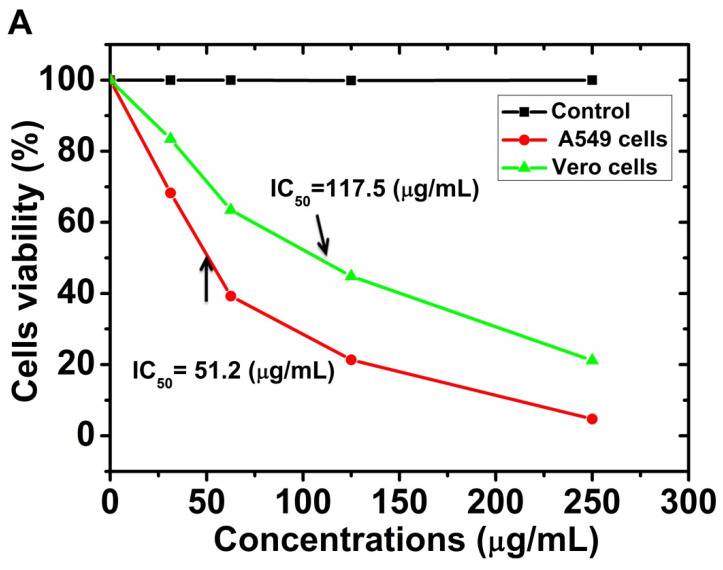

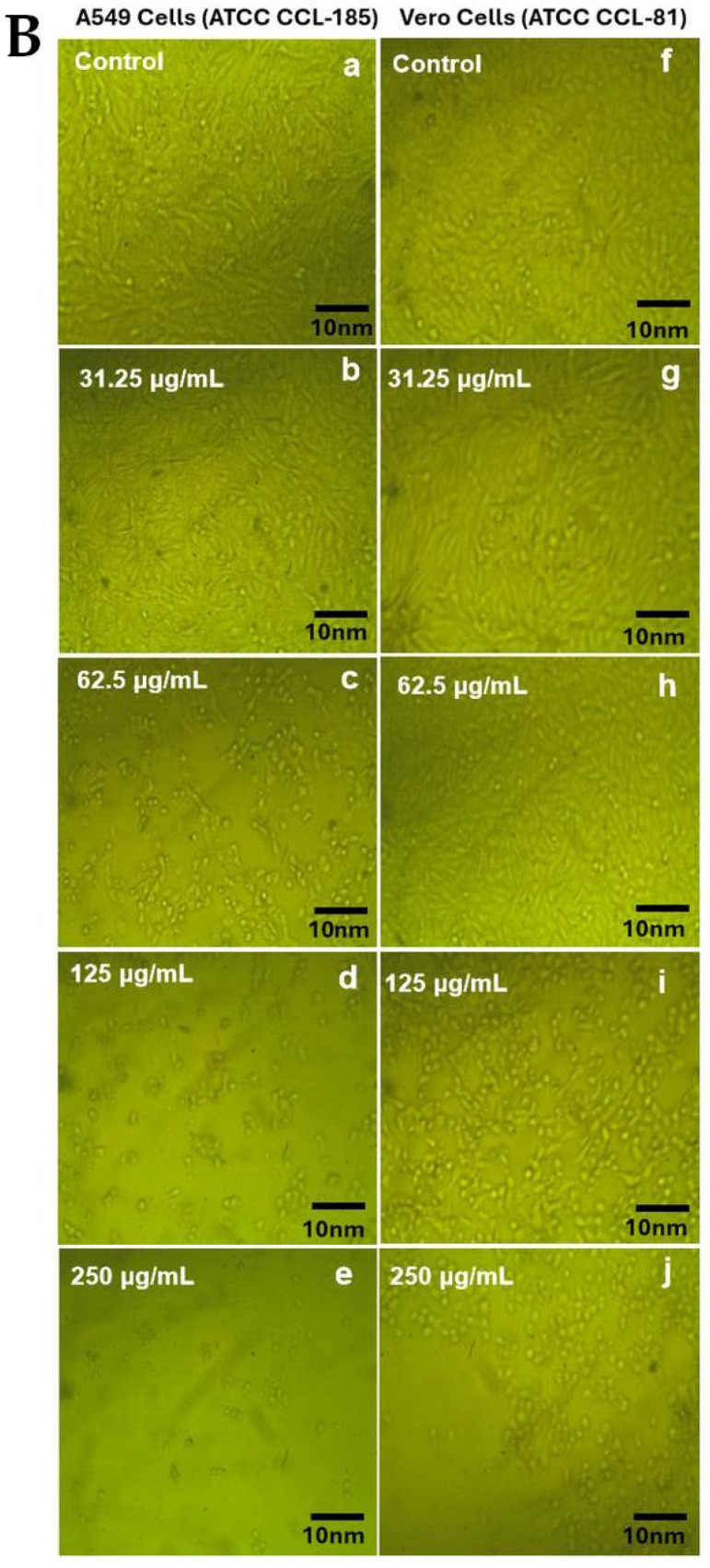
(**A**) Percentage of viable Vero and A549 cells treated with different concentrations of *SRLe-*αFe_2_O_3_ at 48 h in MTT assay. Numerical data are reported as the mean ± SD (*n* = 3). (**B**) In vitro cytotoxicity of NPs on (**a**–**e**) A549 and (**f**–**j**) Vero cell lines (scale bar =10 µm).

The cell viability and cytotoxic effect of different concentrations of *SRLe*-αFe_2_O_3_ nanoparticles at 31.25 to 250 μg/mL was observed against A549 cells and Vero-derived kidney epithelia isolated from green monkeys; the results are shown in Figure 11A,B. The results clearly show that, in the absence of *SRLe*-αFe_2_O_3_NPs synthesized from the green species, 100% of the cell lines survived the damage. The treatment of A549 lung cancer cell lines with *SRLe*-αFe_2_O_3_ NPs inhibited the proliferation of cell lines in a time-dependent and dose-dependent manner. It was observed that the morphology was not effectively destroyed when *SRLe*-αFe_2_O_3_ NPs were used at low concentrations, with a complete loss of cells occurring at higher concentrations [52]. Nevertheless, the synthesized NPs exhibited high toxicity against the A459 cell line, with only 5.4% (IC_50_ = 51.2 μg/mL) of the cells surviving after 48 h. Approximately similar results of synthesized αFe_2_O_3_ nanoparticles using L-ascorbic acid as a reducing agent were reported by Kumar et al. (2022), with an IC_50_ ≤30 µg/mL [53]. These outcomes may be explained by the usage of higher dosages of *SRLe*-αFe_2_O_3_ NPs, leading to excessive production of ROS-mediated oxidative stress in the cell and, thus, DNA damage, as presented in previous investigations [54]. Furthermore, approximately 20–25% (IC_50_ = 117.5 μg/mL) of the Vero cells survived when given the same *SRLe*-αFe_2_O_3_ NPs up to a dose of 250 μg/mL. These results are similar to those of Bhuiyan et al. [50]. This suggests that cancer cells may be more vulnerable to ROS than normal cells and that ROS-mediated processes may be used to target cancer cells [55]. Because iron-based nanoparticles are a potent inducer of ROS, a sufficient quantity can selectively destroy tumor cells while also inhibiting their development. A459 cells are isolated from lung cancer cells; thus, nanoparticles can be utilized to cure cancer, as cancer cells have greater amounts of ROS and more oxidative DNA damage than normal cells in the same regions of tissue. Overall, the results of our investigation confirm that utilizing an aqueous leaf extract of *Stevia rebaudiana* L. to synthesize *SRLe*-αFe_2_O_3_ NPs is an ecofriendly and high-efficiency approach.

The present study had some limitations that need to be addressed in the future. Firstly, the in vitro effect of the designed formulations needs to be evaluated on pre-established biofilms. Secondly, a lack of funding precluded the completion of an antibiotic resistance experiment on both bacteria tested. Future investigations will reveal whether the biosynthesized alpha hematite Fe_2_O_3_ from Stevia leaf extract can exhibit synergism and disruptive effects on biofilm-based infection as a result of its effective antibacterial activity against established in this study. In addition, we recommend undertaking in vivo studies to demonstrate the efficacy of our designed formulations.

## 3. Materials and Methods

### 3.1. Materials

*S. rebaudiana* was obtained from Sugar Crops Research Institute (SCRI, Egypt). Methanol (MeOH), ethyl acetate (EtOAc), chloroform (CHCl_3_), and acetone (AcOH) solvents were purchased from Merck (Darmstadt, Germany). FeCl_3_·6H_2_O and FeCl_2_·4H_2_O were obtained from Aladdin Chemical Reagent Company (Shanghai, China). Folin–Ciocâlteu reagent, 2,2-diphenyl-1-picryl-hydrazyl (DPPH) radicals, and all HPLC standards were purchased from Sigma–Aldrich (Germany). *E. coli* and *S. aureus* were isolated and identified at the Microbiology Unit in the National Cancer Institute Faculty of Medicine Cairo University, Cairo, Egypt.

### 3.2. Preparation of S. rebaudiana Samples

The *S. rebaudiana* materials were washed several times using ddH_2_O, and all parts (leaves, stems, and roots) were dried in the open air at room temperature. Lastly, a powder was obtained by grinding all parts mechanically before storing in polyethylene bags in a freezer at −4 °C until use.

### 3.3. Extraction of Phenolic Compounds from S. rebaudiana

TPCs were extracted following the method of Anokwuru et al. [56] with some modifications. Briefly, 10 g of each dried powder part was soaked in 100 mL of ethyl acetate, acetone, or CHCl_3_/MeOH (2:1 *v*/*v*) in conical flasks for 72 h at room temperature with shaking (Thermo Scientific™, MaxQ™ 420HP). Then, all samples were filtered through Whitman filter paper No 42 and concentrated under reduced pressure using a rotary evaporator in vacuo at 45 °C. Finally, the extracts were preserved in sterilized airtight, labeled bottles in a refrigerator at 4 °C until required for analysis.

### 3.4. Determination of Extraction Yield (EY)

The extraction yield (EY) was calculated as using the following formula:

(1) $$EY\left(\%\right)=\frac{WE}{DW}\times 100$$

where WE is the weight of the extract after evaporating the solvent and freeze-drying, and DW is the dry weight of the sample.

### 3.5. Evaluation of TPC Using Folin–Ciocâlteu Assay

The TPC of the *S. rebaudiana* extracts was defined using the Folin–Ciocâlteu assay [57]. In brief, 200 µL of extract (10 mg/mL) was added to 2.0 mL of a solution of 10 mL of Na_2_CO_3_ (2% *w*/*v*), 0.1 mL of CuSO_4_, and 0.1 mL of sodium and potassium tartrate, before mixing. Then, 0.4 mL of NaOH (0.5 M) was added after 4 min to the mixture, while 0.2 mL of Folin-Ciocâlteu reagent (1:1 *v*/*v*) was added after 10 min. Next, the solution was left for 30 min, and its absorbance was estimated using a UV/Vis spectrophotometer at 750 nm. The TPC was calculated as mM GAE using a GA standard curve.

### 3.6. Evaluation of Antioxidant Activity

The 2,2-diphenyl-1-picryl-hydrazyl (DPPH) radical-scavenging assay was carried out according to a previously reported method [58]. Briefly, 0.4 mL of *Stevia* extract was mixed with 3.6 mL of a methanol solution of DPPH (0.1 mM). An equal amount of 0.4 mL of methanol (0.004% *w*/*v*) was used as a blank with 3 mL of DPPH solution. All samples were evaluated in triplicate, vortexed for 3 min, and incubated in the dark for 35 min at 37 °C. The reduction in absorbance of each sample was measured against methanol as a blank on a UV spectrophotometer (Miltton Roy, Spectronic 1201) at 515 nm, and the data were recorded every minute for 16 min. The PI of the DPPH antioxidant activity was calculated using the following formula:

(2) $$\mathrm{Percentage}\;\mathrm{inhibition}\;\left(\mathrm{PI}\right)=\frac{\mathrm{AC}\;\mathrm{at}\;t\;=0\;\min-\mathrm{AT}\;\mathrm{at}\;t\;=16\;\min\;}{\mathrm{AC}\;\mathrm{at}\;t\;=0\;\min}$$

where AC is the absorbance of the control, and AT is the absorbance of the sample + DPPH. The results were reported as the IC_50_ value, with a lower value representing stronger DPPH scavenging capacity. An ascorbic acid standard curve was used as a positive control.

### 3.7. Evaluation of Total Phenolic Compound Using HPLC Assay

HPLC was carried out using a GBC 1100 Series HPLC system equipped with a UV detector [59]. Bioactive phenolic components were identified in SRLe using a C18 column (250 mm × 4.6 mm; 5 μm). The mobile phase consisted of 10.2% acetic acid in 2 mM sodium acetate (solvent A) and acetonitrile (solvent B). The flow rate was kept constant at 1 mL/min for a total run time of 10 min at 25 °C. The system was run with an isocratic program (70:30 B/A) The injection volume was 50 μL of CHCl_3_/MeOH (2:1 *v*/*v*) extract.

### 3.8. Biosynthesis of SRLe-αFe_2_O_3_ Nanoparticles

*SRLe-*αFe_2_O_3_ NPs were synthesized using ferric chloride (FeCl_3_·6H_2_O), as described in [60]. Briefly, 40 mL of ferric chloride (2 mM) was placed in an Erlenmeyer flask and stirred for more than 1 h. SRLe solution (4 mL) was then added dropwise into the ferric chloride solution with vigorous stirring (200 rpm) at ambient room temperature (25–27 °C) for 4 h to allow the formation of *SRLe-*αFe_2_O_3_. Then, 1 M of NaoH was added until attaining pH 11. The solution eventually turned cloudy black. Subsequently, the solution was centrifuged at 12,000 rpm for 10 min and washed with dH_2_O to remove any impurities or absorbed ions. Finally, the product was dried at 60–70 °C for 48 h using a fan-assisted oven (Figure 1A,B).

### 3.9. SRLe-αFe_2_O_3_ NP Distribution and Characterization

#### 3.9.1. Particle Size (PS) and ζ-Potential (ZP)

The mean PS and ZP of the formulations were measured using DLS (Malvern Instruments, UK). For size estimation, 3 mL of bare *SRLe-*αFe_2_O_3_ NPs were diluted in deionized water, placed in a cell cuvette, and scanned four times to get an average reading. The mean ± SD was obtained after three measurements.

#### 3.9.2. Surface Morphology

The *SRLe-*αFe_2_O_3_ NPs samples were imaged using transmission electron microscopy (TEM; TOPCON002B; Tokyo, Japan). Thin *SRLe-*αFe_2_O_3_ NPs films were created on a carbon-coated copper grid by simply dropping a small quantity of sample on the grid, before blotting away any excess solution using blotting paper [61]. The optimized samples were imaged using scanning electron microscopy (SEM) (JSM 6390^®^, JEOL DATUM Ltd., Tokyo, Japan). A drop of *SRLe-*αFe_2_O_3_ NPs was dried onto an aluminum grid under a mercury lamp for 5 min to obtain a coating thickness of 400 Å.

#### 3.9.3. UV/Visible Spectrometry

The UV/Vis spectra of diluted samples were measured using a Varian Cary-100 Konc spectrophotometer (Varian, Wien, Australia) at 230 V/50 Hz, in the wavelength range of 300–600 nm [62].

#### 3.9.4. X-ray Fdiffraction (XRD)

XRD patterns of the as-synthesized *SRLe-*αFe_2_O_3_ NPs were determined using a Rigaku D/Max-lllC X-ray diffractometer (Rigaku Int. Corp., Tokyo, Japan) at a voltage of 40 kV and a current of 40 kA. The patterns were recorded as a function of the 2θ angle in the range of 10°−80° with a step size of 0.01° at a scanning rate of 0.02 steps/s with the help of a monochromatized X-ray beam with a copper filter (CuKα, λ = 1.54178 Å).

#### 3.9.5. FTIR

The optical properties of *SRLe-*αFe_2_O_3_ NPs were characterized using an FTIR spectrometer (JASCO FT-IR 4100 spectrometer, Hachioji, Tokyo, Japan) to inspect the functional groups contained in the prepared samples. Potassium bromide (KBr) was mixed with the prepared samples. A disc was loaded at high pressure and measured at a wavelength of 400–4000 cm^−1^ with a resolution of 4.0 cm^−1^.

### 3.10. Antimicrobial Activity According to Agar Well Diffusion Assay

The well diffusion method was performed in triplicate according to the method in [63]. Briefly, the bacterial suspension was prepared and spread on Mueller–Hinton agar using a swab and then left for 5 min to dry. Next, five holes were created, with one of the pits containing the standard control erythromycin, while 100 µL of *SRLe-*αFe_2_O_3_ NPs were added to each pit at successive concentrations (5, 10, 15, and 30 mg/mL), before incubating for 24 h at 37 °C. After incubation, zones of growth inhibition were measured to the nearest millimeter to determine the antimicrobial potency of the screened antimicrobial substances [64]. The results are expressed as the mean ± standard deviation (SD).

### 3.11. Cytotoxicity and Anticancer Studies

The cytotoxic assessment of A459 cells derived from lung cancer cells (ATCC CCL-185) and Vero cells isolated from kidney epithelia extracted from African green monkeys (ATCC CCL-81) were evaluated using the MTT assay (5 mg/mL in PBS). The medium from the wells was evacuated after incubation. Then, MTT (20 µL) was incorporated into each well along with 25 μL of *SRLe*-αFe_2_O_3_ NPs (autoclaved). The cells were dissolved in 200 µL of DMSO (dimethyl sulfoxide). The absorbance spectra of the specimens were distinguished by recording the optical density at 560 nm and subtracting the background at 620 nm using a microplate reader [65].

(3) $$\%\;\mathrm{Cell}\;\mathrm{viability}\;=\frac{\mathrm{OD}\;\mathrm{test}\;-\;\mathrm{OD}\;\mathrm{blank}}{\mathrm{OD}\;\mathrm{control}\;-\;\mathrm{OD}\;\mathrm{blank}}$$

where test denotes the cells exposed to the *SRLe*-αFe_2_O_3_ NP sample, control denotes the control sample, and blank denotes the wells without Vero cells and A549 cells [66].

### 3.12. Statistical Analysis

Each experiment was carried out at least in triplicate, and all data were presented as the mean ± SD. Analysis of statistical significance was performed using one-way ANOVA and the post-hoc Tukey test (*p* < 0.05). All analysis was conducted using SAS 9.4 for Windows x64 from the SAS Institute (Cary, North Carolina), and graphical outputs were generated using GraphPad Prism software (Version 8, GraphPad Software Inc., San Diego, CA, USA).

## 4. Conclusions

This study focused on the green synthesis of alpha hematite αFe_2_O_3_ NPs from *S. rebaudiana* leaf extract, which was efficaciously implemented as an antimicrobial and anticancer agent. DLS, UV/Vis, XRD, EDX, and FTIR analyses were used to determine the features, size, shape, and thermal stability of *SRLe*-αFe_2_O_3_ NPs nanoparticles, while TEM and SEM microscopic methods were utilized to detect the morphological qualities of the surfaces of the green synthesized nanoparticles. The results showed that the *SRL* extract with CHCl_3_/MeOH (2:1 *v*/*v*) (least polar solvent) contained the highest EY and TPC of ~3.5% and ~75 mg GAE/g extract, respectively, along with an antioxidant radical-scavenging activity of IC_50_ = 12.87 ± 0.7 mg/mL. These phenolic compounds played an important role in increasing the stability of *SRLe*-αFe_2_O_3_ NPs. The antibacterial efficacy of synthesized NPs toward isolated Gram-negative and Gram-positive bacteria was moderate. Although nanoparticles were toxic at high concentrations, they demonstrated remarkable effectiveness (eliminating almost 94% of cancer cells) against the A549 lung cancer cell line, indicating that they might be a viable choice for eradicating tumor cells at optimal doses. However, further research is needed to discover the exact doses and reaction conditions needed to employ nanoparticles for these purposes.

## Figures and Tables

**Table 1:** Effect of different solvents on EY and TPC for *SRLe*.

Samples	AcOH		CHCl_3_/MeOH (2:1 *v*/*v*)		EtOAc	
	EY %	TPC (mg REs/g)	EY %	TPC (mg REs/g)	EY %	TPC (mg REs/g)
Leaves	1.27 ± 0.02	18.77 ± 0.61	3.52 ± 0.05	74.79 ± 0.62	2.78 ± 0.7	64.75 ± 0.59
Stems	0.51 ± 0.03	11.75 ± 0.57	2.75 ± 0.04	47.6 ± 0.09	2.61 ± 0.04	43.34 ± 0.04
Roots	2.21 ± 0.03	55.65 ± 0.05	1.93 ± 0.25	21.45 ± 0.04	0.91 ± 0.06	7.06 ± 0.03

Vertical bars illustrate the SD (*n* = 3).

**Table 2:** HPLC Chromatogram of bioactive compounds (Rt = 10 min) of CHCl_3_/MeOH (2:1 *v*/*v*) sample extract of *SRLe* (dried leaves to solvent, *w*/*v*), along with the molecular formula, molecular weight, and biological properties.

Bioactive Phenolic Compounds	PubChem CID	Molecular Formula	Molecular Weight	Biological Properties	Concentration (µg/mL)	RT
Gallic acid	370	C_7_H_6_O_5_	170.12	Antioxidant activity assessed as DPPH free-radical-scavenging activity	13.483	1.7
Chlorogenic acid	1794427	C_16_H_8_O_4_	354.31	Inhibition of HDAC in Hela cell nuclear extracts according to fluorometric assay	0.195	3
Caffeic acid	689043	C_9_H_8_O_4_	180.16	Antioxidant activity assessed as DPPH free-radical-scavenging activity	0.231	3.6
Coumaric acid	637542	C_9_H_8_O_3_	164.16	Inhibition of human CA2 according to stopped-flow CO2 hydration assay	6.154	4.5
Ferulic acid	445858	C_10_H_10_O_4_	194.18	Antioxidant and antiproliferative activity against human MCF7 cells	3.587	5.5
Protocatechuic acid	72	C_7_H_16_O_4_	154.12	Antioxidant activity assessed as DPPH free-radical-scavenging activity	1.247	6.1
Catechin	9064	C_15_H_14_O_6_	290.27	Antioxidant and noncompetitive inhibition of Leishmania amazonensis recombinant arginase expressed in *E. coli* Rosetta (DE3) pLysS	0.731	7.6
Syringic acid	10742	C_9_H_10_O_5_	198.17	Antioxidant activity assessed as DPPH free-radical-scavenging activity	7.825	8.6

## Data Availability

Not applicable.
