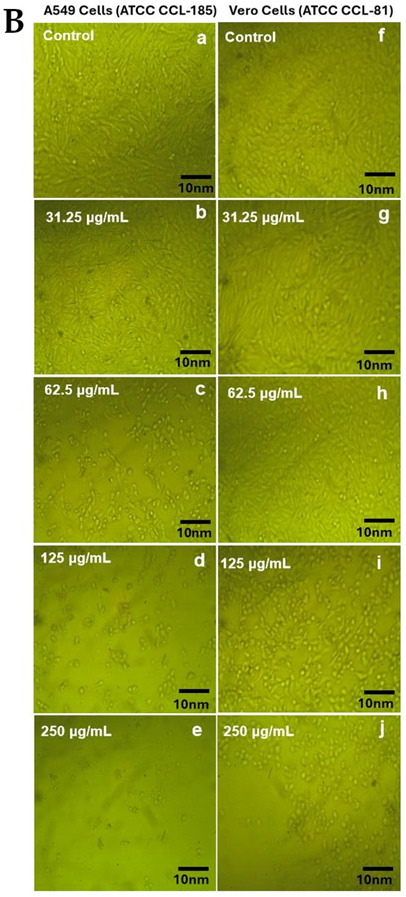# Correction: Alshawwa et al. In Situ Biosynthesis of Reduced Alpha Hematite (α-Fe_2_O_3_) Nanoparticles by *Stevia Rebaudiana* L. Leaf Extract: Insights into Antioxidant, Antimicrobial, and Anticancer Properties. *Antibiotics* 2022, *11*, 1252

**DOI:** 10.3390/antibiotics14070690

**Published:** 2025-07-08

**Authors:** Samar Zuhair Alshawwa, Eman J. Mohammed, Nada Hashim, Mohamed Sharaf, Samy Selim, Hayaa M. Alhuthali, Hind A. Alzahrani, Alsayed E. Mekky, Mohamed G. Elharrif

**Affiliations:** 1 Department of Pharmaceutical Sciences, College of Pharmacy, Princess Nourah bint Abdulrahman University, Riyadh 11671, Saudi Arabia; szalshawwa@pnu.edu.sa; 2 Department of Biology, College of Science, Mustansiriyah University, Baghdad 14022, Iraq; emanjassim@uomustansiriyah.edu.iq; 3 General Practitioner, Faculty of Medicine, University of Gezira, Wad Medani 318, Sudan; hashimnada345@gmail.com; 4 Department of Biochemistry, Faculty of Agriculture, AL-Azhar University, Cairo 11751, Egypt; 5 Department of Clinical Laboratory Sciences, College of Applied Medical Sciences, Jouf University, Sakaka 72341, Saudi Arabia; 6 Department of Clinical laboratory sciences, College of Applied Medical Sciences, Taif University, Taif 21944, Saudi Arabia; hmhuthali@tu.edu.sa; 7 Basic Sciences, Applied Medical Sciences, Albaha University, Albaha 4781, Saudi Arabia; hindalzahrani@bu.edu.sa; 8 Department of Botany and Microbiology, Faculty of Science, Al-Azhar University, Cairo 11884, Egypt; alsayedessam@azhar.edu.eg; 9 Department of Basic Medical Sciences, College of Medicine, Shaqra University, Shaqra 11961, Saudi Arabia; al_harrif@yahoo.com

In the original publication [1], there was a mistake in Figure 11B. Figure 11Bf and Figure 11Bh were inadvertently duplicated. The corrected Figure 11B is provided below. The authors state that the scientific conclusions are unaffected. This correction was approved by the Academic Editor. The original publication has also been updated.